# Supplementary material for: Time‑resolved multi-omic analysis of paclitaxel exposure in human iPSC‑derived sensory neurons unveils mechanisms of chemotherapy‑induced peripheral neuropathy
Source: Cell Death Dis. 2026 Feb 10;17(1):211. doi: 10.1038/s41419-026-08445-2 (PMC12921266; doi:10.1038/s41419-026-08445-2)
Supplement: Supplementary file 15 — Dataset 1. [file 41419_2026_8445_MOESM15_ESM.zip › CDD_Open_Data/04_RNA_Seq/Timeline_panel_plots_etc_2023-01-23_1609.zip!/Panel_Plots_etc/Evidence_Plots/48h/evidence_plot_standard contrasts_msigdb_reactome_48h_PTX_vs_DMSO_ID5_M27172_REACTOME_ACTIVATION_OF_GENE_EXPRESSION_BY_SREBF_SREBP.pdf]

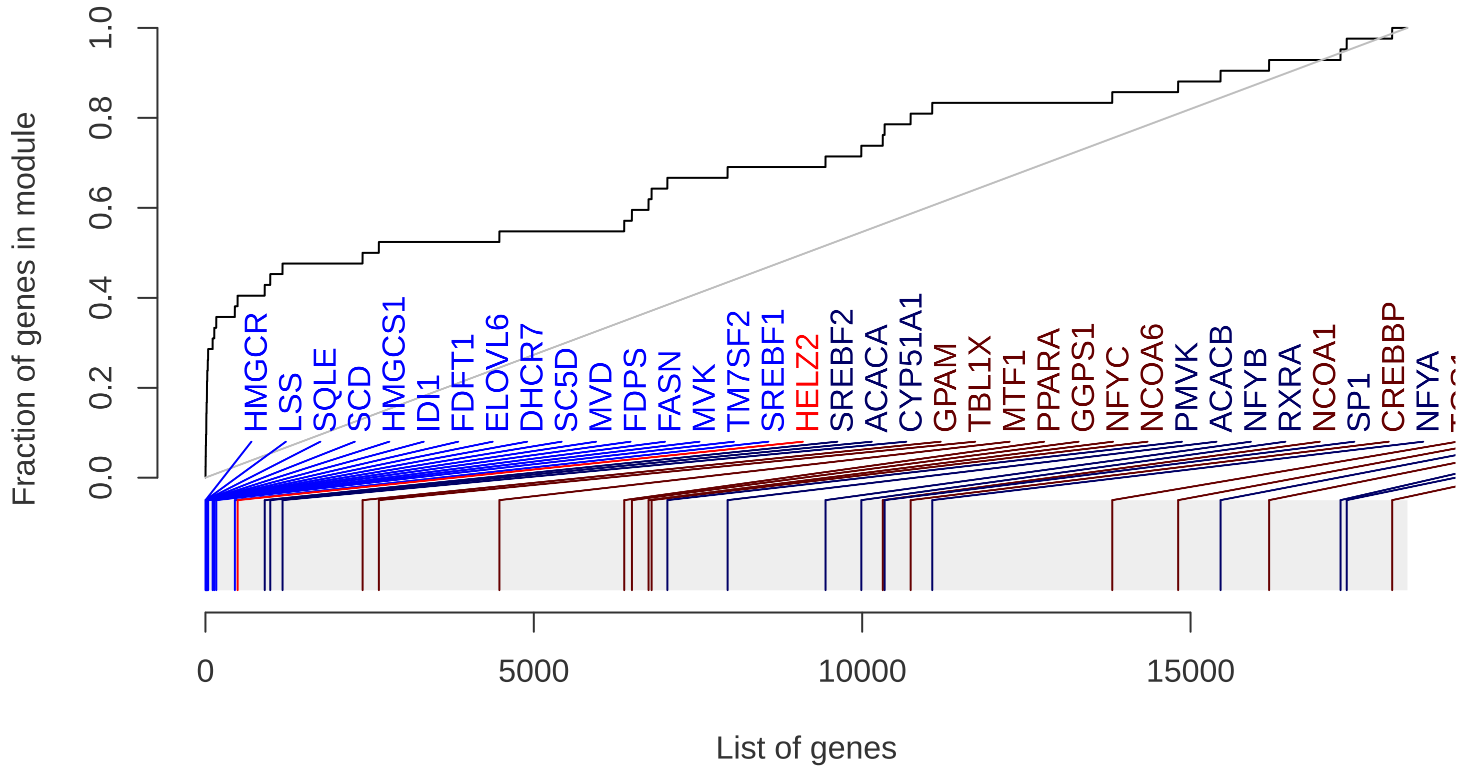

Fraction of genes in module
0.0
0.2
0.4
0.6
0.8
1.0
HMGCR
LSS
SQLE
SCD
HMGCS1
IDI1
FDFT1
ELOVL6
DHCR7
SC5D
MVD
FDPS
FASN
MVK
TM7SF2
SREBF1
HELZ2
SREBF2
ACACA
CYP51A1
GPAM
TBL1X
MTF1
PPARA
GGPS1
NFYC
NCOA6
PMVK
ACACB
NFYB
RXRA
NCOA1
SP1
CREBBP
NFYA
0
5000
10000
15000
List of genes
